# Functional analyses of epidemic *Clostridioides difficile* toxin B variants reveal their divergence in utilizing receptors and inducing pathology

**DOI:** 10.1371/journal.ppat.1009197

**Published:** 2021-01-28

**Authors:** Zhenrui Pan, Yuanyuan Zhang, Jianhua Luo, Danyang Li, Yao Zhou, Liuqing He, Qi Yang, Min Dong, Liang Tao

**Affiliations:** 1 Key Laboratory of Structural Biology of Zhejiang Province, School of Life Sciences, Westlake University, Hangzhou, China; 2 Center for Infectious Disease Research, Westlake Laboratory of Life Sciences and Biomedicine, Hangzhou, China; 3 Institute of Basic Medical Sciences, Westlake Institute for Advanced Study, Hangzhou, China; 4 Department of Urology, Boston Children’s Hospital, Boston, Massechusetts, United States of America; 5 Department of Surgery and Department of Microbiology, Harvard Medical School, Boston, Massechusetts, United States of America; University of Texas Medical School at Houston, UNITED STATES

## Abstract

*Clostridioides difficile* toxin B (TcdB) is a key virulence factor that causes *C*. *difficile* associated diseases (CDAD) including diarrhea and pseudomembranous colitis. TcdB can be divided into multiple subtypes/variants based on their sequence variations, of which four (TcdB1-4) are dominant types found in major epidemic isolates. Here, we find that these variants are highly diverse in their receptor preference: TcdB1 uses two known receptors CSPG4 and Frizzled (FZD) proteins, TcdB2 selectively uses CSPG4, TcdB3 prefers to use FZDs, whereas TcdB4 uses neither CSPG4 nor FZDs. By creating chimeric toxins and systematically switching residues between TcdB1 and TcdB3, we determine that regions in the N-terminal cysteine protease domain (CPD) are involved in CSPG4-recognition. We further evaluate the pathological effects induced by TcdB1-4 with a mouse intrarectal installation model. TcdB1 leads to the most severe overall symptoms, followed by TcdB2 and TcdB3. When comparing the TcdB2 and TcdB3, TcdB2 causes stronger oedema while TcdB3 induces severer inflammatory cell infiltration. These findings together demonstrate divergence in the receptor preference and further lead to colonic pathology for predominant TcdB subtypes.

## Author summary

*Clostridioides difficile* is a major cause of nosocomial and community-associated gastrointestinal infections. The bacterium produces three exotoxins including TcdA, TcdB, and CDT, of which TcdB is known as a key virulence factor causing the diseases. Since *C*. *difficile* was first linked to antibiotic-associated infections in 1978, a large number of clinically relevant strains were characterized and many of them were found to harbor some variant forms of TcdB. In this study, we examined four predominant TcdB variants from epidemic *C*. *difficile* strains. We found that these variants are highly diverse in preference to the known receptors, CSPG4 and Frizzled proteins. By conducting a systematically designed mutagenesis study, we determined that TcdB interacts with CSPG4 via regions across multiple domains. We also found that TcdB variants could induce distinguishable pathological phenotypes in a mouse model, suggesting *C*. *difficile* strains harboring divergent TcdB variants might exhibit different disease progression. Our study provides new insights into the toxicology and pathology of *C*. *difficile* toxin variants.

## Introduction

*Clostridioides difficile* (formerly known as *Clostridium difficile*) is a spore-forming anaerobic bacterium that is a major cause of nosocomial and community-acquired gastrointestinal infections. Once the normal gut flora is disrupted by antibiotic treatment, *C*. *difficile* can colonize the colon and induce diarrhea and pseudomembranous colitis [1,2]. Due to the emergence of hypervirulence and antibiotic-resistant strains, the global burden of *C*. *difficile* infection (CDI) is exacerbated [3–5].

Two homologous exotoxins, TcdA (308 kDa) and TcdB (270 kDa), are the major virulence factors produced by *C*. *difficile* that disrupt the colonic epithelium and induce tissue damage. TcdB is considered as the primary disease-causing toxin because TcdB alone can induce a full spectrum of disease in both animals and humans [6–8], and many TcdA–TcdB+ strains have been clinically isolated [9,10]. Some *C*. *difficile* strains also express a third toxin called *C*. *difficile* binary toxin (CDT), which has been reported to suppress the host eosinophilic response [11].

TcdB is a single-chain exotoxin belonging to the large clostridial toxin family. It consists of four functional domains: an N-terminal glucosyltransferase domain (GTD), a cysteine protease domain (CPD) that mediates auto-cleavage, a central domain (DRBD) responsible for both transmembrane delivery and receptor-binding, and a C-terminal combined repetitive oligopeptides (CROPs) domain. Like other large clostridial toxins, TcdB enters the host cells via receptor-mediated endocytosis and glucosylates small GTPase proteins, leading to actin depolymerization, cell rounding, and eventually cell death [12–14].

Although the prototypical TcdB (TcdB1) is a major form expressed in the clinical *C*. *difficile* isolates such as VPI10463 and 630, natural variants of TcdB are widespread and can be grouped into 8 subtypes/variants [15]. Notably, some variants are expressed in strains particularly important to the epidemiology of CDI. For instance, major outbreaks of CDI associated with hypervirulent ST1/RT027 strains emerged in North America from 2001 to 2007 and rapidly spread to other places in the world [3,16–19]. These lineages express a TcdB variant (TcdB2), which contains ~7.9% sequence variation from TcdB1, mainly within the C-terminal half of DRBD and the CROPs domain [20]. Another example is that the predominant *C*. *difficile* strains in Asia, ST37/RT017 [21–24], express a TcdB variant (TcdB3) that contains an overall ~6.4% sequence variation from TcdB1. Recently, Quesada et al. reported a new TcdB variant (TcdB4) [25], which is potentially a result of recombination between TcdB2, TcdB3, and TcdB7.

Progress has been made recently in defining protein receptors for members of the large clostridial toxin family including TpeL [26] from *Clostridium perfringens*, TcdA [27] and TcdB [28–30] from *C*. *difficile*, and TcsL [31,32] from *Paeniclostridium sordellii*. For TcdB, three candidate receptors have been characterized including chondroitin sulfate proteoglycan 4 (CSPG4) [28,30], poliovirus receptor-like 3 (PVRL3) [29], and the Wnt receptor frizzled proteins (FZDs, particularly FZD1, 2, and 7) [30]. FZDs are the major receptors in colonic epithelium while CSPG4 possibly mediates the toxin entry in the intestinal subepithelial myofibroblasts layer [30,33]. While CSPG4 and FZDs can mediate the toxin binding/entry that resulting in cytopathic cell-rounding effects at low toxin concentrations, PVRL3 was shown only to be involved in the cytotoxicity of TcdB at high toxin concentrations[29]. As soluble PVRL3 failed to protect either cell lines and intestinal organoids from TcdB [30,34], whether and how PVRL3 contributes to the cytopathic toxicity remains to be elucidated.

The molecular basis of TcdB-FZD interaction has been established by a recent co-crystal structural study [33]. TcdB binds to the ectodomain of FZD2 via a region between residues 1420–1630 and uses a fatty acid docked within FZD2 as the co-receptor. Binding of TcdB not only mediates toxin entry but also directly inhibits the Wnt-signaling pathways [30,33,35]. The molecular mechanisms of TcdB-CSPG4 and TcdB-PVRL3 interactions remain to be further established at structural levels. Previous studies suggested that PVRL bound TcdB in a CROPs-independent manner [36] and the junction region between the DRBD and CROPs is critical for CSPG4 binding [37]. In addition to these protein receptors, the CROPs domain in TcdB might also bind to carbohydrates on cell surfaces [38].

Although CSPG4 and FZDs are well established as cellular receptors for TcdB1, it remains to be examined whether they are universally utilized by other TcdB subtypes, particularly those epidemically important ones. For instance, a key residue (F1597) that mediates TcdB1-FZD2 interaction is altered to serine in TcdB2 [33,39]. It was previously reported that TcdB2 showed loss of binding to FZD2 *in vitro* [39,40], albeit it remains to be functionally examined at the cellular level whether TcdB2 can utilize FZDs and/or CSPG4 as its receptors. These findings indicate that sequence variations in TcdB subtypes may potentially alter their receptor recognition preferences.

Here, we investigate the contribution of CSPG4 and FZD1/2/7 in mediating the toxin binding/entry of clinically important TcdB1-4 variants by utilizing CSPG4 and FZD1/2/7 knockout HeLa cells. We found that TcdB1-4 have different preferences in utilizing CSPG4 and FZDs as cellular receptors. Moreover, we identified key residues within the CPD that are critical to mediating CSPG4-recognition. These findings suggest the CSPG4-binding interface could be composed of discontinuous regions from multiple TcdB domains that spatially converge. Importantly, we found that TcdB1-4 exhibit differences in inducing pathological phenotypes in the mice colon tissue, suggesting *C*. *difficile* strains harboring divergent TcdB variants may exhibit distinct pathological progresses.

## Results

### TcdB variants from epidemic *C*. *difficile* differ in receptor preference

TcdB1-4 are major variants that together were found in over 99.6% of the pathogenic *C*. *difficile* strains [15]. To determine the functional contribution of CSPG4 and FZD1/2/7 in mediating the toxin entry of these TcdB variants into host cells, we compared the activities of TcdB variants using the standard cytopathic cell-rounding assay on the HeLa-Cas9 (parental cell line, referred to as the wildtype (WT) HeLa thereafter) cells versus CSPG4 knockout (*CSPG4*^*–/–*^) and FZD1/2/7 knockout (*FZD1/2/7*^*–/–*^) HeLa cells, which were generated previously via CRISPR/Cas9 approach [30]. The toxin concentration that induces 50% of cells to become round is defined as CR_50_, which is routinely used to compare the sensitivities of different cells to TcdB variants. HeLa cells express a high level of CSPG4, low levels of FZDs, and probably no PVRL3 [30,37]; they are suitable to investigate the preference of TcdB variants on CSPG4 and FZDs.

**Fig 1:**
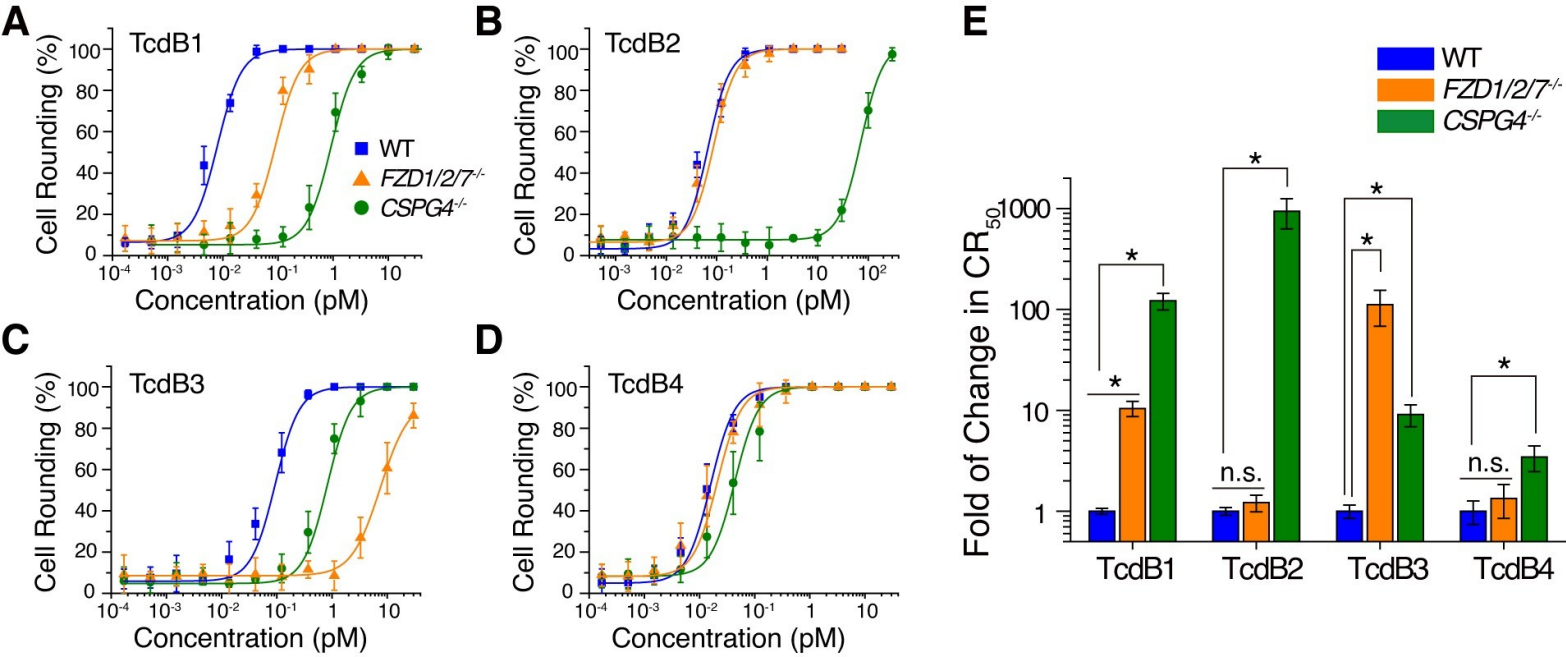
TcdB variants have different receptor preference in HeLa cells. (A-D) The sensitivities of HeLa wildtype (WT), *FZD1/2/7*^*–/–*^, and *CSPG4*^*–/–*^cells to TcdB1 (A), TcdB2 (B), TcdB3 (C), and TcdB4 (D) were measured using the cytopathic cell-rounding experiments (n = 6). (E) The change of toxin resistance in different cell lines was quantified and normalized to the WT. Error bars indicate mean ± standard deviation. (n = 6, *P<0.001, n.s. = not significant, versus the WT, student’s *t-*test.).

TcdB1 (reference sequence from *C*. *difficile* strains 630) was previously utilized for the identification of cellular receptors including CSPG4 and FZDs. As expected, both the *CSPG4*^*–/–*^and *FZD1/2/7*^*–/–*^cells were more resistant to TcdB1 than the WT cells, which validated that our assay conditions can resolve the individual contributions from CSPG4 and FZD1/2/7. When compared to the WT cells, the *CSPG4*^*–/–*^cells showed higher increased resistance to TcdB1 compared to the *FZD1/2/7*^*–/–*^cells (~100-fold for *CSPG4*^*–/–*^and ~10-fold for *FZD1/2/7*^*–/–*^, Fig 1A and 1E), which is likely because CSPG4 is abundantly expressed in HeLa cells [30,37].

The HeLa WT and *FZD1/2/7*^*–/–*^cells showed similar sensitivity (CR_50_ at ~0.05 pM) towards TcdB2 (reference sequence from strain CD196), indicating that FZD1/2/7 may not contribute to TcdB2 entry into HeLa cells. In contrast, the *CSPG4*^*–/–*^cells showed drastically increased resistance to TcdB2 (~1000-fold), suggesting that CSPG4 is a dominant and likely sole protein receptor for TcdB2 in HeLa cells (Fig 1B and 1E).

TcdB3 (reference sequence from strain 1470) also showed reduced potency on both *CSPG4*^*–/–*^and *FZD1/2/7*^*–/–*^cells, but the *FZD1/2/7*^*–/–*^cells showed much higher increased resistance to TcdB3 compared to the *CSPG4*^*–/–*^cells (~10-fold for *CSPG4*^*–/–*^and ~100-fold for *FZD1/2/7*^*–/–*^, Fig 1C and 1E). These results suggest that, although TcdB3 can utilize both CSPG4 and FZD1/2/7 as receptors, FZD1/2/7 are its dominant receptors, despite that CSPG4 is highly expressed in HeLa cells.

Surprisingly, TcdB4 (reference sequence from strain 8864) equally affected both the WT and *FZD1/2/7*^*–/–*^cells and showed only a minimal reduction (~3-fold) on the *CSPG4*^*–/–*^cells compared with the WT cells, which is drastically different from TcdB1-3 (Fig 1D and 1E).

### TcdB2 does not utilize FZD1/2/7 as its cellular receptors

**Fig 2:**
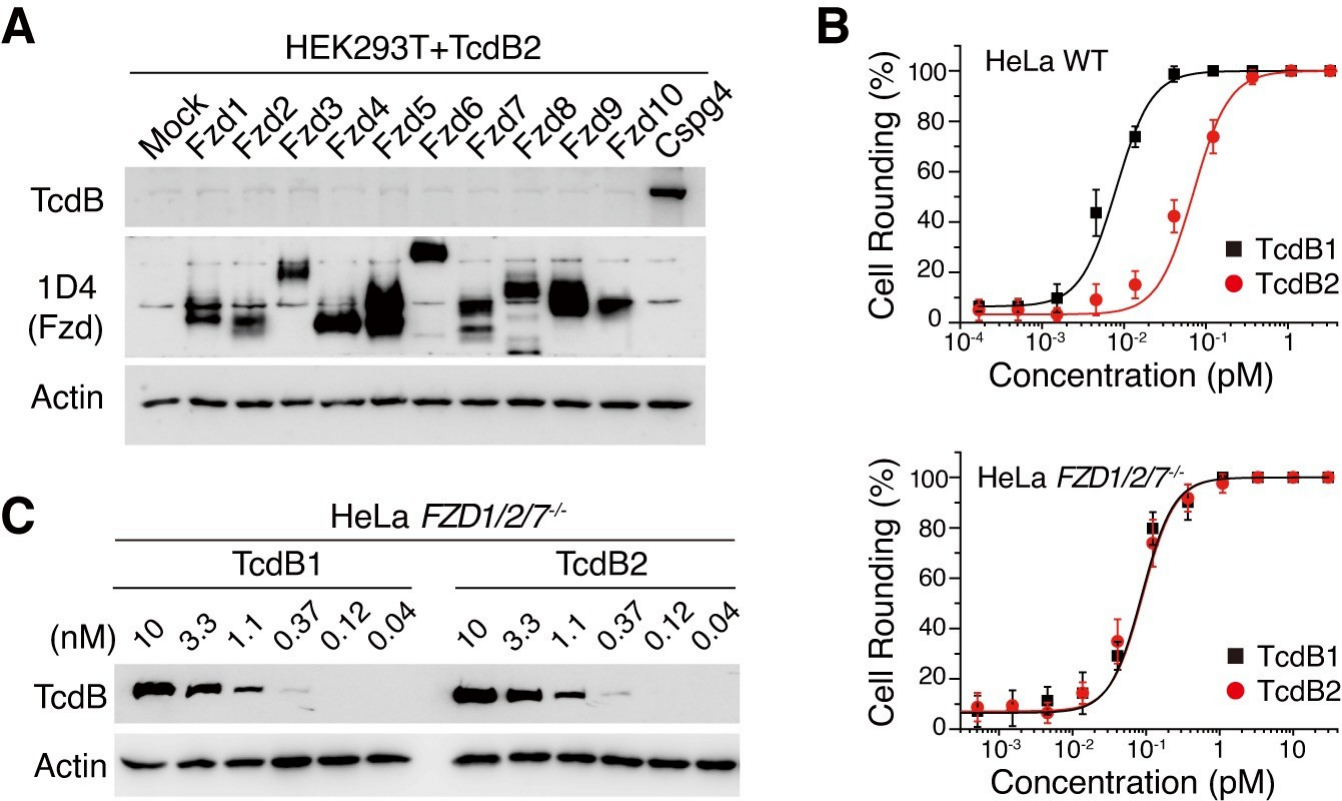
TcdB2 loses binding to ten Frizzled members. (A) Transfection of rat Cspg4 but not ten mouse Frizzled proteins increased TcdB2 binding to HEK293T cells, assayed by immunoblot analysis. (B) HeLa WT and *FZD1/2/7*^*–/–*^cells were tested for sensitivity towards TcdB1 and TcdB2. The percentage of rounded cells were plotted over toxin concentrations. (Error bars indicate mean±s.d., n = 6) (C) Surface binding of TcdB1 and TcdB2 on HeLa *FZD1/2/7*^*–/–*^cells, assayed by immunoblot analysis.

To study the FZD1/2/7-binding and CSPG4-binding of TcdB1, TcdB2, and TcdB3 at the cellular level, we utilized a polyclonal anti-TcdB antibody, which recognizes different TcdB subtypes including TcdB1, TcdB2, and TcdB3 with similar efficacy (S1 Fig), to measure the toxin binding on the surface of cells hereafter. To further confirm that TcdB2 binds CSPG4 but not FZDs on cell surfaces, we measured binding of TcdB2 to HEK293T cells transiently expressing mouse Fzd homologs (Fzd1-10) or rat Cspg4. None of the overexpressed FZD homologs increased the cell surface binding of TcdB2, whereas over-expression of the rat Cspg4 resulted in the robust binding of TcdB2 (Fig 2A). These results demonstrated that TcdB2 not only lost binding to FZD1/2/7 but also failed to bind other frizzled family member proteins.

TcdB2 (CR_50_ at ~0.05 pM) is about 9-fold less potent than TcdB1 (CR_50_ at ~0.006 pM) in the HeLa WT cells and both TcdB1 and TcdB2 showed similar toxicity (CR_50_ at ~0.06 pM) to *FZD1/2/7*^*–/–*^cells (Fig 2B), suggesting that the CSPG4-binding abilities of TcdB1 and TcdB2 are similar to each other while TcdB2 specifically loses binding to FZD1/2/7. We further measured the cell surface binding of TcdB1 and TcdB2 on HeLa *FZD1/2/7*^*–/–*^cells, of which CSPG4 remains the sole receptor. As expected, TcdB2 had equivalent or slightly better bindings to the *FZD1/2/7*^*–/–*^cells compared to TcdB1 (Fig 2C), suggesting CSPG4 mediates a similar level of cell surface binding for TcdB1 and TcdB2.

### TcdB3 has reduced binding to CSPG4

**Fig 3:**
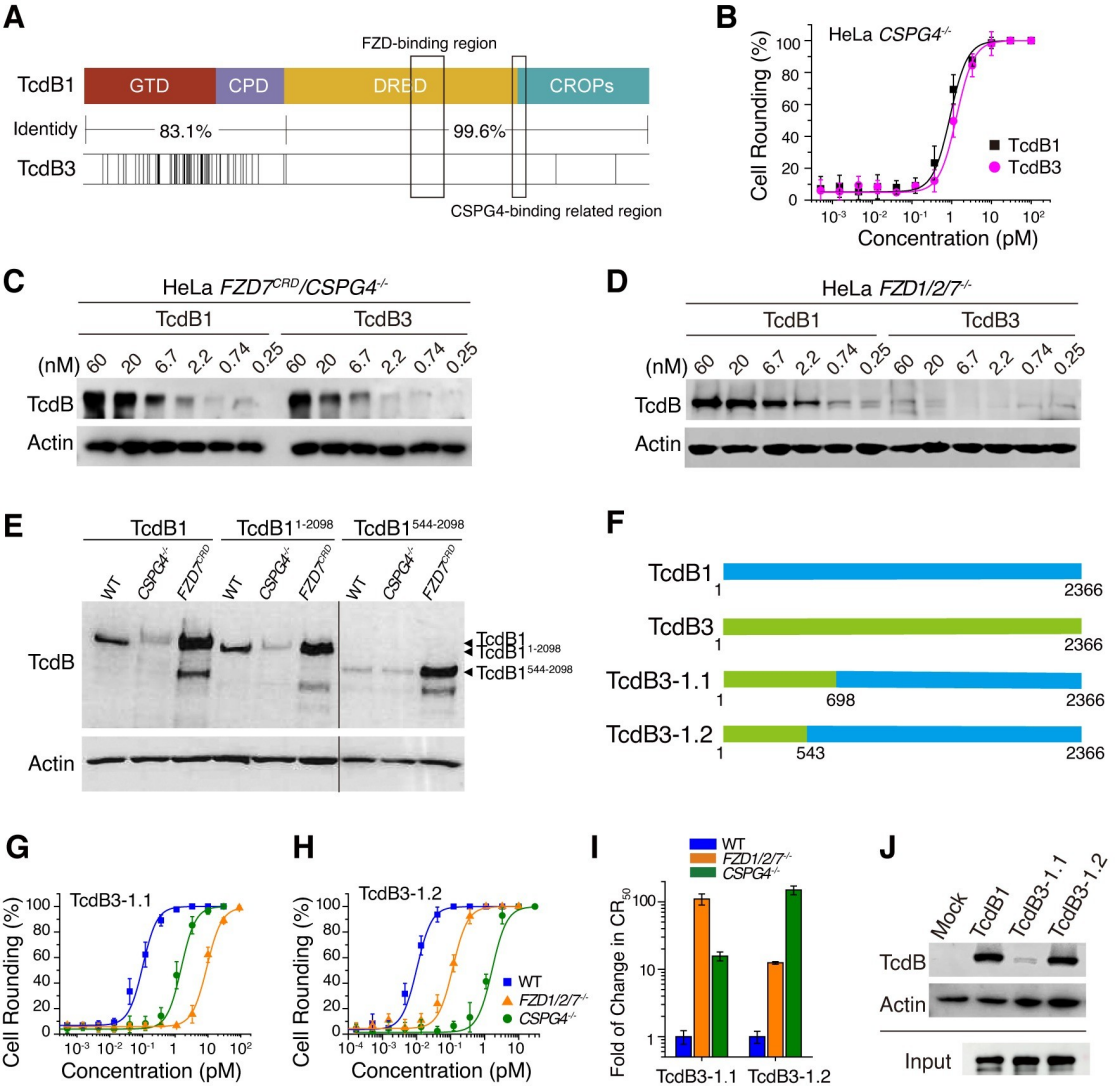
TcdB3 has reduced binding to CSPG4. (A) Sequence alignment shows that TcdB1 and TcdB3 are highly conserved at the C-terminal half (identity of 99.6), but less conserved in the GTD and CPD (identity of 83.1%). The primary sequences of TcdB1 and TcdB3 in the FZD binding region and previously identified CSPG4-binding related region 1 are the same. (B) HeLa *CSPG4*^*–/–*^cells were tested for the sensitivity towards TcdB1 and TcdB3. The percentage of rounded cells were plotted over toxin concentrations. (Error bars indicate mean±s.d., n = 6) (C) The surface binding experiment showed that TcdB1 and TcdB3 had similar levels of binding to the HeLa *FZD7*^*CRD*^*/CSPG4*^*–/–*^cells, assayed by immunoblot analysis. (D) The surface binding experiment showed that TcdB3 had significantly decreased binding to the HeLa *FZD1/2/7*^*–/–*^cells compared to TcdB1, assayed by immunoblot analysis. (E) The surface binding experiment showed that full-length TcdB1 and TcdB1^1-2098^ robustly bound to the HeLa WT cells but not to the CSPG4 knockout cells. TcdB1^544-2098^ weakly bound to both HeLa WT and *CSPG4*^*–/–*^cells. HeLa cells overexpressing the ectodomain of FZD7 (*FZD7*^*CRD*^) were used as the positive control and all three proteins strongly bound to these cells. (F) Schematic drawing of designed chimeric toxins based on TcdB1 and TcdB3. (G-I) The sensitivities of the HeLa WT, *FZD1/2/7*^*–/–*^, and *CSPG4*^*–/–*^cells to TcdB3-1.1 (G) and TcdB3-1.2 (H). The percentage of rounded cells were plotted over toxin concentrations. Their CR50 were measured and plotted in a bar chart (I). (Error bars indicate mean±s.d., n = 6) (J) The surface binding experiment showed that TcdB1 and TcdB3-1.2, but not TcdB3-1.1 robust bound to the *FZD1/2/7*^*–/–*^cells, assayed by immunoblot analysis.

The primary sequences of TcdB1 and TcdB3 are mainly different in the GTD and CPD, while the identity in the DRBD and CROPs between TcdB1 and TcdB3 is ~99.6% (Fig 3A). In particular, TcdB1 and TcdB3 share the almost identical amino acid sequence for the FZD-binding region. There is one divergent amino acid (position 1575) and this residue is far away from the TcdB-FZD interface according to the structure (Figs 3A and S2). Indeed, we observed that the toxicity of TcdB1 and TcdB3 were close to each other in the HeLa *CSPG4*^*–/–*^cells (Fig 3B), indicating that TcdB1 and TcdB3 have similar ability to utilize FZD1/2/7 as their receptors.

Because the expression levels of endogenous FZDs are low in HeLa cells, we previously generated the HeLa cells expressing high levels of the GPI-anchored ectodomain of FZD7 on the surface (HeLa *FZD7*^*CRD*^) that enables robust FZD-mediated binding of TcdB for immunoblot analysis [33]. Based on these cells, here we further generated the HeLa *FZD7*^*CRD*^/*CSPG4*^*–/–*^cells with CSPG4 knocked-out via CRISPR/Cas9 technique and compared the binding of TcdB1 and TcdB3 to the HeLa *FZD7*^*CRD*^/*CSPG4*^*–/–*^and *FZD1/2/7*^*–/–*^cells.

TcdB3 showed similar binding to TcdB1 on *FZD7*^*CRD*^/*CSPG4*^*–/–*^cells (Fig 3C) but virtually no detectable binding to *FZD1/2/7*^*–/–*^cells, whereas TcdB1 showed robust concentration-dependent binding to *FZD1/2/7*^*–/–*^cells (Fig 3D). These data suggest that TcdB3 has a drastically reduced ability to interact with CSPG4 but maintains similar binding to FZD7 on cell surfaces compared with TcdB1, which is consistent with the low fold-of-change in the sensitivity of *CSPG4*^*–/–*^cells to TcdB3 compared with WT cells (Fig 1E).

### GTD and CPD contribute to TcdB-CSPG4 interaction

Previous studies showed that truncating the entire CROPs domain disrupt CSPG4-binding [30], whereas keeping only a short stretch of the N-terminal part of the CROP domain is sufficient to preserve the binding of CSPG4 [37]. It was also shown that point mutations at the junction region between the DRBD and CROPs can disrupt the binding of CSPG4 [37], further suggesting that this region is involved in CSPG4-binding. However, sequence alignment reveals that this region is highly conserved between TcdB1 and TcdB3 (Fig 3A), raising the possibility that other regions of TcdB are also involved in CSPG4-binding.

Notably, TcdB1 is particularly distinct from TcdB3 in the first 850 amino acids which consist of the GTD and CPD with an identity of only 83.1% (Fig 3A). To determine the regions involved in CSPG4-binding, we generated three truncated toxins, TcdB1^1-2098^, TcdB1^544-2098^, and TcdB1^1285-2366^, and tested their binding to HeLa WT and *CSPG4*^*–/–*^cells (Figs 3E and S3). TcdB1^1-2098^ and full-length TcdB showed similar levels of binding to WT cells, and their binding was greatly reduced on *CSPG4*^*–/–*^cells, demonstrating that these binding events are mediated by CSPG4 and do not require the C-terminal part of the CROPs domain (Fig 3E). In contrast, TcdB1^544-2098^ showed only residual weak binding to both WT and *CSPG4*^*–/–*^cells at similar levels (Fig 3E). Cells stably overexpress the GPI-anchored ectodomain of FZD7 showed robust binding of TcdB1^544-2098^, TcdB1^1-2098^, and full-length TcdB1 to cells, demonstrating that TcdB1^544-2098^ was well-folded and maintained FZD-mediated binding but specifically loses CSPG4-mediated binding. Consistently, TcdB1^1285-2366^ also showed only residual weak binding to the WT and *CSPG4*^*–/–*^cells at similar levels (S3 Fig).

To further investigate whether GTD and/or CPD of TcdB contribute to CSPG4-binding, we next generated two TcdB1/TcdB3 chimeric toxins designated TcdB3-1.1, which contains the residues 1–698 from TcdB3 and residues 699–2366 from TcdB1, and TcdB3-1.2, which contains residues 1–543 from TcdB3 and residues 544–2366 from TcdB1, as illustrated in Fig 3F. We measured the cytopathic effects induced by these chimeric toxins in the HeLa WT, *CSPG4*^*–/–*^, and *FZD1/2/7*^*–/–*^cells. Interestingly, the cell rounding experiments showed that the toxicities of TcdB3-1.1 and TcdB3 to the WT cells are almost identical (~0.08 pM versus ~0.07 pM for CR_50_, S4A Fig). TcdB3-1.1 is also similar to TcdB3 as it showed ~100-fold reduced toxicity to the *FZD1/2/7*^*–/–*^cells and ~15-fold reduced toxicity to the *CSPG4*^*–/–*^cells (Fig 3G and 3I). On the other hand, the toxicity of TcdB3-1.2 and TcdB1 to the WT cells are close to each other (~0.008 pM versus ~0.006 pM for CR_50_, S4B Fig). TcdB3-1.2 is also resembling TcdB1 as it showed higher increased resistance to the *CSPG4*^*–/–*^cells (~150-fold) than the *FZD1/2/7*^*–/–*^cells (~10-fold, Fig 3H and 3I). Consistent with the cytopathic experiments, TcdB1 and TcdB3-1.2, but not TcdB3-1.1, showed robust binding to *FZD1/2/7*^*–/–*^cells (Fig 3J). These results indicate that the sequence differences within the residues 543–698 between TcdB1 and TcdB3, which are located in the CPD, are likely responsible for their differences in CSPG4-binding.

### Mutagenesis screens identify key residues in CPD for CSPG4-binding

**Fig 4:**
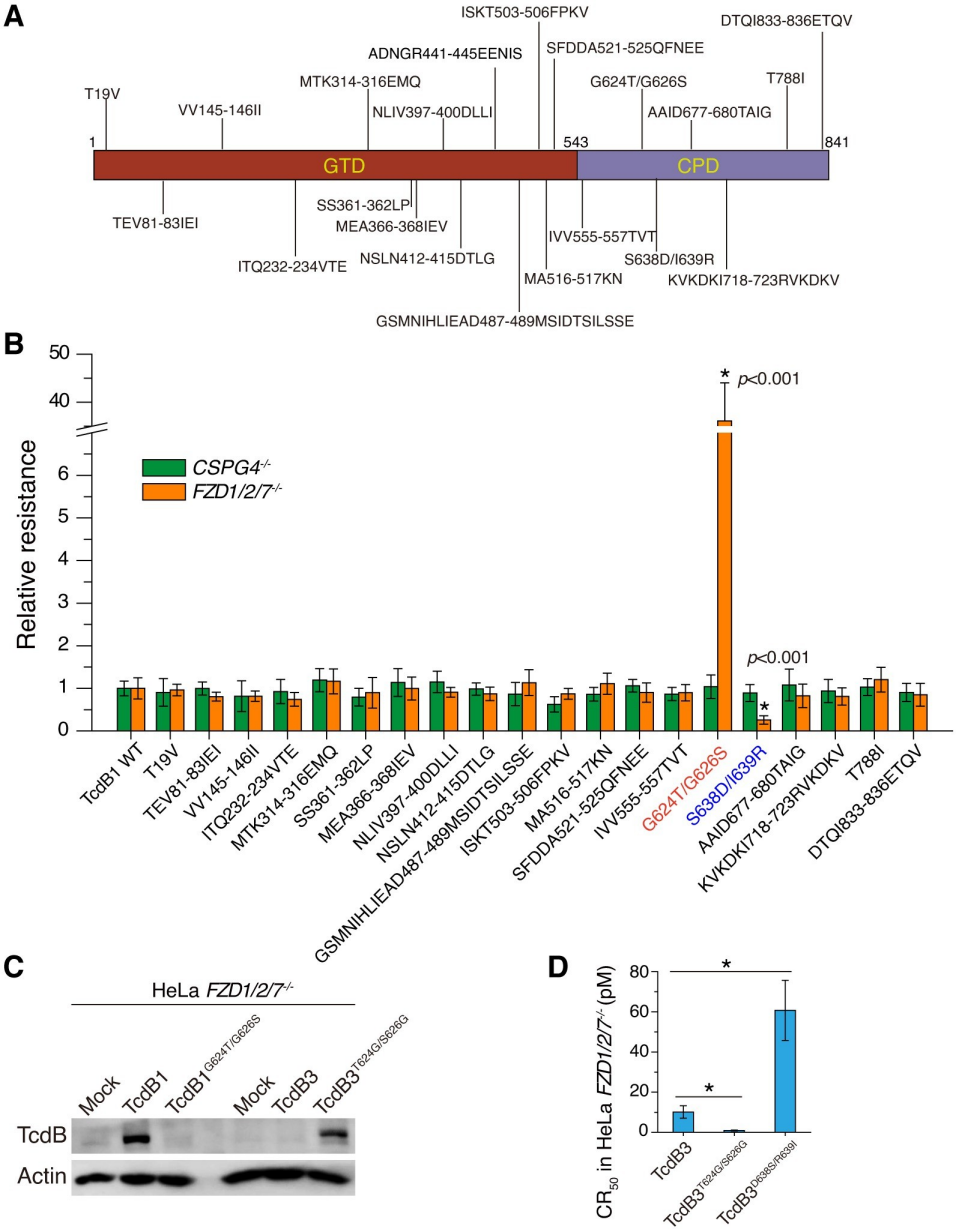
Mutagenesis screens identify residues critical for CSPG4-binding. (A) Schematic drawing of mutations created based on the sequence divergence between TcdB1 and TcdB3. (B) Effects of the sequence substitutions in TcdB1 on cytopathic assays in the HeLa *CSPG4*^*–/–*^and *FZD1/2/7*^*–/–*^cells. Changes of the relative resistance of 20 mutants compared to the WT TcdB1 were shown in a bar chart. G624T/G626S showed drastically decreased toxicity and S638D/I639R showed increased toxicity in the HeLa *FZD1/2/7*^*–/–*^cells. (Error bars indicate mean±s.d., n = 6, *P<0.001 versus the WT, student’s *t-*test.) (C) Surface binding on the *FZD1/2/7*^*–/–*^cells showed that TcdB1^G624T/G626S^ had decreased binding compared with TcdB1 and TcdB3^T624G/S626G^ had increased binding compared with TcdB3, assayed by immunoblot analysis. (D) The CR_50_ of TcdB3, TcdB3^T624G/S626G^, TcdB3^D638S/R639I^ were measured and plotted in a bar chart. (Error bars indicate mean±s.d., n = 6, *P<0.001 versus TcdB3, student’s *t-*test.).

To further determine whether there are specific residual changes within the N-terminus of TcdB3 that are responsible for the reduction in CSPG4-binding, we sought to screen residues that are different between TcdB1 and TcdB3 within their GTD-CPD domain. In total, twenty-one groups of TcdB1 mutants were generated by systematically replacing the residues in TcdB1 with the corresponding residues in TcdB3 (Fig 4A). The activities of these mutant toxins were assessed using cell-rounding assays and compared with WT TcdB1 on both HeLa *CSPG4*^*–/–*^and *FZD1/2/7*^*–/–*^cells (S5 Fig). Among the twenty-one mutant toxins, one mutant (ADNGR441-445EENIS) showed reduced potency (~50-fold) on both *CSPG4*^*–/–*^and *FZD1/2/7*^*–/–*^cells compared with WT TcdB1 (S6 Fig); these residue changes may have disrupted the folding/function of TcdB1. The other twenty mutant toxins all showed similar levels of potency on the *CSPG4*^*–/–*^cells comparable to WT TcdB1 (Figs 4B and S5), demonstrating that the toxin activities other than CSPG4-binding were not altered by any of these residue changes. Eighteen out of these twenty mutants also showed similar levels of toxicity on *FZD1/2/7*^*–/–*^cells comparable to WT TcdB1, indicating that these residue changes do not impact CSPG4-mediated toxin binding and entry. Two mutant toxins stand out: G624T/G626S and S638D/I639R, with their ability to intoxicate the *FZD1/2/7*^*–/–*^cells changed (Fig 4B).

TcdB1^G624T/G626S^ is approximately 35-fold less toxic than the WT TcdB1 by measuring the cytopathic effect (Fig 4B). To investigate whether the replacement of G624T/G626S directly affects the TcdB1 binding to CSPG4, we performed the cell surface binding experiment with TcdB1 and TcdB1^G624T/G626S^. TcdB1 showed strong binding to the *FZD1/2/7*^*–/–*^cells but the binding of TcdB1^G624T/G626S^ was drastically reduced in both immunoblot and immunofluorescence analyses (Figs 4C and S7A). We further studied the impact of G624T and G626S on TcdB1 toxicities and found that both mutations reduced the potency of TcdB1 on *FZD1/2/7*^*–/–*^cells (~4-fold and ~15-fold, S7B Fig). We also introduced the mutations T624G/S626G, which mimics TcdB1, into TcdB3. As expected, TcdB3^T624G/S626G^ was more potent (~11-fold) and showed increased binding to the *FZD1/2/7*^*–/–*^cells compared to the WT TcdB3 based on immunoblot analysis (Fig 4C and 4D).

TcdB1^S638D/I639R^ is slightly more potent (~3-fold) than the WT toxin in the *FZD1/2/7*^*–/–*^cells (Fig 4B). To further validate that amino acid positions 638 and 639 in TcdB contribute to CSPG4-recognition, we introduced the mutations D638S/R639I into TcdB3 and this mutant toxin was indeed less toxic (~6-fold) than WT TcdB3 in the *FZD1/2/7*^*–/–*^cells (Fig 4D).

### TcdB interacts with CSPG4 via regions across multiple domains

**Fig 5:**
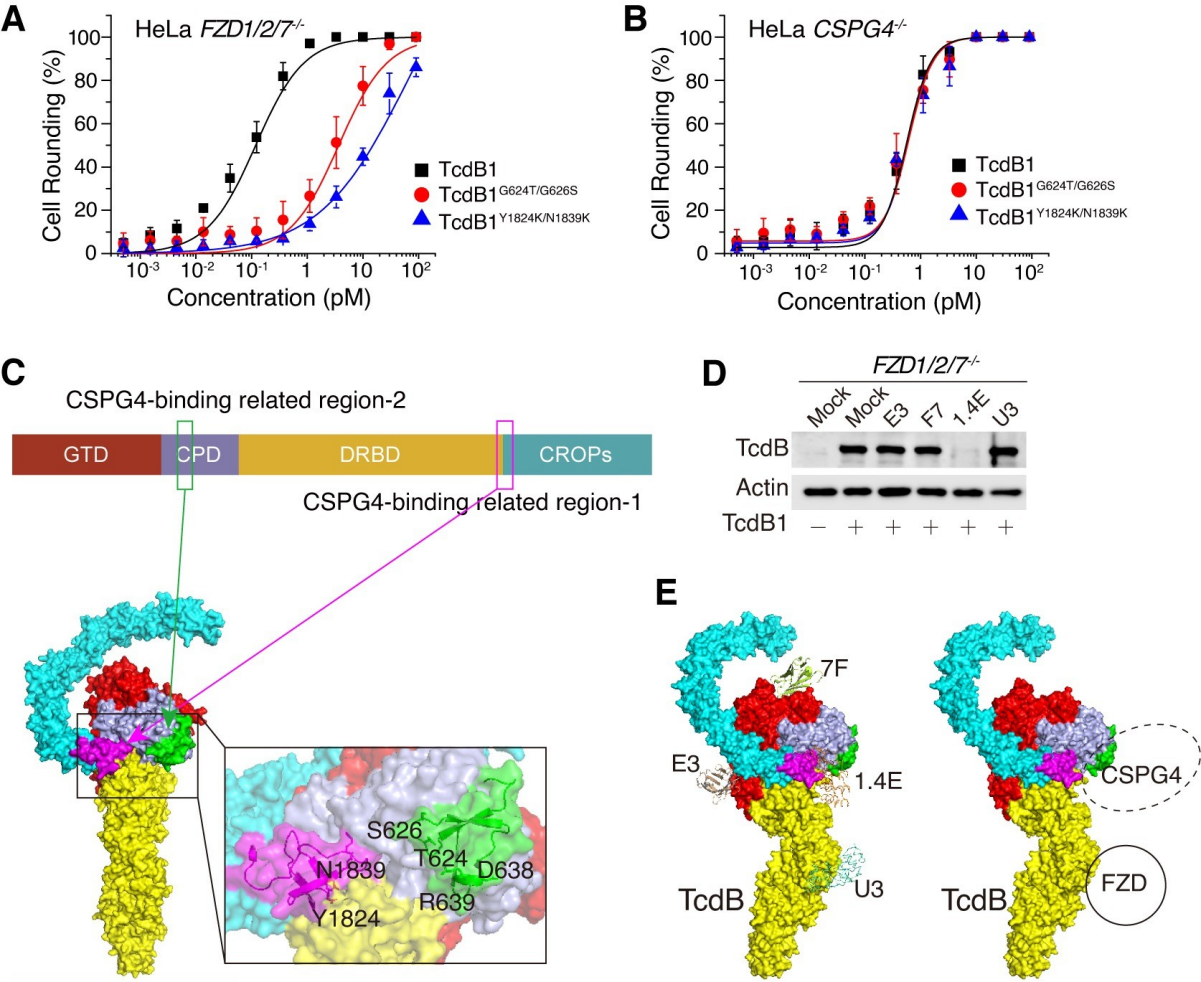
TcdB binds to CSPG4 via regions apart in multiple domains. (A-B) The sensitivities of the HeLa *FZD1/2/7*^*–/–*^(A) and *CSPG4*^*–/–*^(B) cells to TcdB1, TcdB1^G624T/G626S^, and TcdB1^Y1824K/N1839K^. The percentage of rounded cells were plotted over toxin concentrations. (Error bars indicate mean±s.d., n = 6) (C) Schematic drawing and cartoon representations of two CSPG4-binding related regions in TcdB. The previously proposed CSPG4-binding related region-1 (CBR1) is highlighted in magenta and the newly defined CSPG4-binding related region-2 (CBR2) is highlighted in green. (D) Competition assay showed that 1.4E but not E3, F7, and U3 could effectively block the binding of TcdB1 to the HeLa cell surface. (E) Cartoon representations of the proposed model of TcdB-CSPG4 binding.

The previous studies suggested that the junction region (designated as CSPG4-binding related region-1, or CBR1) between the DRBD and CROPs is required for CSPG4 binding and point mutations at residues Y1824 and N1839 disrupt toxin binding to CSPG4 [37]. We also validated that TcdB1^Y1824K/N1839K^ was much less toxic than WT TcdB1 in the HeLa *FZD1/2/7*^*–/–*^cells (Fig 5A), while both WT TcdB1 and TcdB1^Y1824K/N1839K^ showed similar levels of toxicity to the HeLa *CSPG4*^*–/–*^cells (Fig 5B). As our experiments demonstrated that amino acid positions 624, 626, 638, and 639 are important for TcdB-CSPG4 interactions, we mapped these residues onto the recently resolved structure of the full-length TcdB [41], which revealed a second potential CSPG4-binding related region (CBR2). CBR1 and CBR2 are over 1,000 residues apart on the primary sequence, but they are spatially close to each other in the 3-D structure with both located at an area where CPD, DRBD, and CROPs converge [41]. Thus, CBR1 and CBR2 possibly could be parts of an integrated interface to mediate CSPG4-binding (Fig 5C). We also modeled the structure of TcdB1 based on PDB 6OQ5 using HHPRED with MODELLER interface and then put the mutations G624T and G626S in [42]. Residues G624 and G626 located at an open loop on the toxin surface, switching these two residues to Thr and Ser did not change the steric conformation of other residues including Y1824 and N1839 (S8 Fig).

Interestingly, this converged region also contains the binding site for a designed ankyrin repeat protein (DARPin) known as 1.4E, which interacts with residues 747–751, 1749–1767, and 1800–1834 of TcdB and reduces binding to CSPG4 *in vitro*^41^. We found that 1.4E can largely block binding of TcdB1 to *FZD1/2/7*^*–/–*^cells (Fig 5D), whereas other small protein binders such as nanobodies E3 and F7, and another DARPin U3, which are known to bind the GTD or the FZD-binding site [41,43], did not affect TcdB binding to *FZD1/2/7*^*–/–*^cells (Fig 5D), demonstrating that 1.4E likely docks into the same interface where CSPG4 binds (Fig 5E). These results further illustrated the importance of the region where CPD, DRBD, and CROPs converged for CSPG4-recognition.

### Predominant TcdB subtypes show differences in colonic pathology

**Fig 6:**
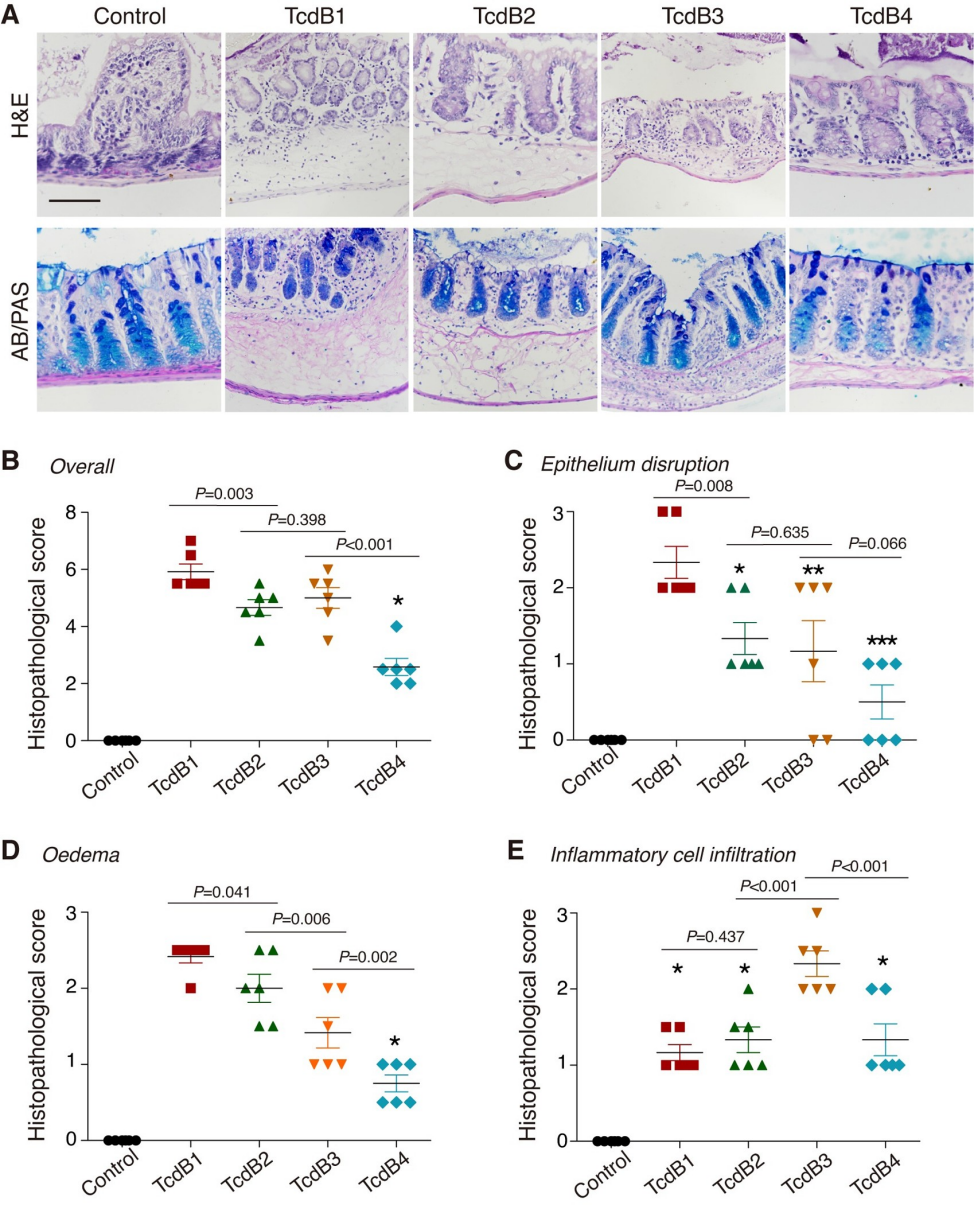
Predominant TcdB subtypes induce distinguishable colonic pathology. (A) Mouse colonic tissues harvested after intrarectal instillation assays were assessed for the pathology induced by TcdB1-4 through H&E staining and AB/PAS staining. (Scale bar, 100 μm.) (B-E) The stained sections were analyzed (combining both H&E and AB/PAS) and histological scores for overall pathology (B), epithelium disruption (C), oedema (D), and inflammatory cell infiltration (E) were given blindly by two pathologists. (n = 6 mice, error bars indicate mean±s.d. *P<0.001 versus control, **P = 0.002 versus control, ***P = 0.161 versus control, one-way ANOVA.).

We next characterized the pathological effects induced by major TcdB variants in the colon using the mouse intrarectal instillation assay, which has been previously established to examine the tissue damage caused by *C*. *difficile* toxins [44,45]. At eight hours post-injection, colonic tissues were dissected out from the mice and subjected to histopathological analysis. As expected, intrarectally injecting 10 μg of TcdB1-4 resulted in damages to the mouse colonic epithelium, including disruption of the epithelial barrier, oedema, and infiltration of inflammatory cells in both mucosa and submucosa of the colon, as showed by both the hematoxylin and eosin (H&E) and Alcian blue/periodic acid-Schiff (AB/PAS) staining (Fig 6A).

Histological scoring (based on assessing the integrity of epithelial barrier, oedema, and infiltration of inflammatory cells) revealed that TcdB1 induced overall the strongest colonic pathology followed by TcdB2 and TcdB3, while TcdB4 only caused mild symptoms (Fig 6B). When analyzed separately, disruption of epithelium follows the same ranking of the overall histological scores, with TcdB1 causing the most severe disruption and TcdB2 and TcdB3 causing similar levels of disruption (Fig 6C). TcdB1 and TcdB2 caused similar degrees of oedema, which are clearly higher than the levels caused by TcdB3 (Fig 6D). In contrast, TcdB3 caused a higher level of inflammatory cell infiltration than both TcdB1 and TcdB2 (Fig 6E). TcdB4 caused mild oedema, moderate inflammatory cell infiltration, and almost no epithelium disruption (Fig 6C–6E).

### The difference in cellular entry of TcdB subtypes leads to divergent pathology

**Fig 7:**
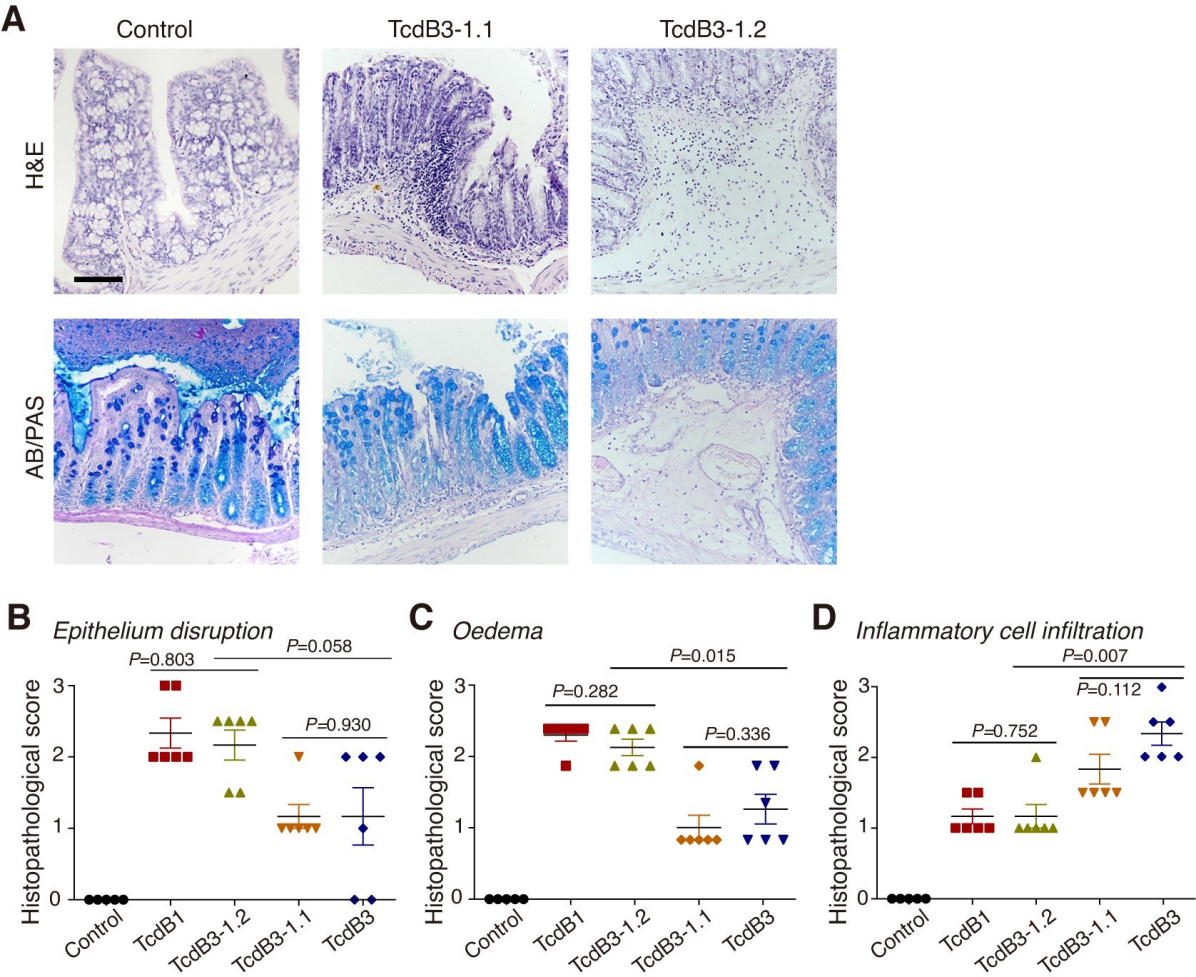
Receptor preference but not substrate specificity determines pathology. (A) Mouse colonic tissues were assessed for the pathology induced by TcdB3-1.1 and TcdB3-1.2 through H&E staining and AB/PAS staining. (Scale bar, 100 μm.) (b-e) The stained sections were analyzed and histological scores for epithelium disruption (B), oedema (C), and inflammatory cell infiltration (D) were given blindly by two pathologists, and then compared to TcdB1 and TcdB3. (n = 6 mice, error bars indicate mean±s.d., two-sided Mann-Whitney test.).

TcdB3 contains a TcsL-like GTD, of which the substrate profile is in part different from TcdB1 and TcdB2 [46,47]. To resolve whether the contrasting pathologic manifestations were due to the receptor preference or substrate specificity of TcdB subtypes, we took the advantages of the two chimeric toxins (TcdB3-1.1 contains the first 698 residues from TcdB3 and the rest part from TcdB1 whileTcdB3-1.2 contains the first 543 residues from TcdB3 and the rest part from TcdB1) and examined their induced pathology in the mouse colon as showed by both H&E and AB/PAS stains (Fig 7A). TcdB3-1.2 induced severe epithelium disruption and oedema, and moderate inflammatory cell infiltration, which was similar to TcdB1. TcdB3-1.1 caused severe inflammatory cell infiltration and moderate epithelium disruption and oedema, which resembled TcdB3 (Fig 7B–7D). These pathologic effects are in line with the defined receptor preference pattern (Fig 3G–3I). Notably, TcdB1 and TcdB3-1.2 contain different GTD but induced similar pathology, while TcdB3 and TcdB3-1.2 contain the same GTD but induced divergent pathology (Fig 7B–7D). These results together suggest that the receptor preference but not the substrate specificity of TcdB determines the pathologic manifestations in the mouse intrarectal instillation model.

## Discussion

TcdB is the major virulence factor responsible for CDI associated diseases (CDAD). The primary sequences of TcdB variants/subtypes are highly divergent. For instance, the sequence difference between TcdB2 and TcdB3 is over 10% [15]. These sequence divergence may lead to variations of certain biological properties such as enzymatic activity, immunogenicity, and receptor recognition [25,39,40,43,46,48–52], which could potentially lead to differences in CDAD manifestation and progression. Here, we reported that the four major TcdB variants from clinical *C*. *difficile* strains showed distinct preferences to use the two defined receptors and induced distinguishable pathological phenotypes in the mouse colon tissue.

Previous studies have reported that TcdB2 loses binding to FZD1/2/7 *in vitro* [34,40]. Here we validated that TcdB2 not only lost binding to FZD1/2/7, but also failed to bind other FZD members on cell surfaces. Utilizing *FZD1/2/7*^*–/–*^and *CSPG4*^*–/–*^cells, we further extended these findings to functional levels and found that TcdB2 showed no reduction in toxicity in *FZD1/2/7*^*–/–*^cells and greatly reduced potency in *CSPG4*^*–/–*^cells. These findings confirm that TcdB2 loses the ability to use FZDs as cellular receptors, but still utilizes CSPG4 as a dominant cellular receptor.

Several previous studies have compared the toxicities between TcdB1 and TcdB2 on multiple cell lines: while TcdB1 showed higher potency in some cells, TcdB2 seemed to be more toxic in the others [39,48,50]. This inconsistency could partially be explained by the different expression levels of CSPG4 and FZDs in various cell lines [30], the difference in auto-processing [49], and potentially additional receptors. Moreover, many cell lines used in those studies, such as CHO and 3T3, were derived from different animal sources. Unlike Frizzled proteins, Cspg4 homologs are less conserved among divergent animals. For example, the primary sequence identity between human CSPG4 and hamster Cspg4 is only 84.5%. TcdB1 and TcdB2 may have different affinities towards Cspg4 from different animals.

Although TcdB3 has a partially different substrate profile compared to TcdB1 and TcdB2 [47], the comparison of the bioactivity between TcdB1 and TcdB3 can be assessed using the cytopathic cell rounding assays. After the examination of the sensitivities of the HeLa WT, *CSPG4*^*–/–*^, and *FZD1/2/7*^*–/–*^cells to TcdB1 and TcdB3, we found that TcdB3 had a functional deficiency to intoxicate cells mediated by CSPG4. The cell surface binding experiments showed that this deficiency was due to greatly reduced binding of TcdB3 to CSPG4. This result was surprising because TcdB3 and TcdB1 shared the same amino acid sequence within the regions previously known to be involved in CSPG4-binding. The highest sequence variations between TcdB1 and TcdB3 are in the GTD and the first half of CPD [46,53]. A potential contribution of GTD and CPD to receptor-binding has never been examined previously. By working with the N-terminally truncated toxins, we clearly showed that both GTD and CPD were essential for the robust toxin binding on the cell surface. We further took advantage of sequence divergence between TcdB1 and TcdB3 and carried out comprehensive mutagenesis screens to interrogate the critical positions involved in the CSPG4-TcdB binding. Our functional screens demonstrate that the region within CPD is critical for CSPG4-binding and identified two double mutation sites, G624T/G626S and S638D/I639R, that drastically impacted CSPG4-binding. The computational modeling suggested that G624T/G626S mutations only affected the local structure of TcdB. G624T/G626S mutations introduce amino acid side chains into the cavity between the CPD and CSGP4 binding residues thus it remains a possibility that these mutations may hinder the interaction through steric interference. These findings demonstrate that CSPG4-binding involves a region within CPD, which we defined as CRB2.

We further propose a potential TcdB-CSPG4 interface where GTD, CPD, and DRBD converge, which is also supported by the competition assay with DARPin 1.4E. This area is distinct from the TcdB-FZD interface, which is consistent with the previous finding that the binding of CSPG4 and FZDs to TcdB are independent [30,34].

TcdB4 barely uses either CSPG4 or FZD1/2/7 as the cellular receptor. TcdB4 and TcdB2 share the same serine residue at position 1597, which could interrupt the palmitoleic acid-mediated TcdB-FZD binding. Also, the sequence identity between TcdB4 and TcdB3 in the first 678 residues is 99.1%, containing the critical residues S626/T624 that likely disturb the CSPG4-binding. Surprisingly, TcdB4 is highly potent to the HeLa WT, *CSPG4*^*–/–*^, and *FZD1/2/7*^*–/–*^cells with the CR_50_ around 0.01–0.04 pM, suggesting a possibility that this variant may use unknown receptor(s) other than CSPG4 and FZD1/2/7 to enter the host cells.

The high diversity of receptor preference may potentially benefit the pathogen on its host adaption. Although the driven force remains unknown, TcdB appears to be rapidly evolving [15] which may provide flexibility to attack a wide range of host cells and tissues. On the other hand, natural toxin variants provide optimal tools to study their contributions to disease progressions. TcdB1 is mostly found in the clinical *C*. *difficile* strains such as ST54/RT012 and ST3/RT001. TcdB2 and TcdB3 are both important variants from epidemic *C*. *difficile* isolates; relevant strains like ST1/RT027 (TcdB2) and ST37/RT017 (TcdB3) account for ~15–30% and ~10–40% of CDI in North America and East Asia respectively [3,17–19,21–24,54,55]. TcdB4 was recently reported and appears to be also widely distributed [15,25], albeit its relevance to the pathology and epidemiology remains to be established. TcdB3 is mostly (if not all) found in the TcdA-negative strains, suggesting this variant is capable of inducing CDAD alone. The receptor is a key factor in determining the vulnerability of host cells/tissues to a pathogen. Previous studies showed that FZD1/2/7 are the major receptors mediating the toxin binding/entry to disrupt the colonic epithelium for TcdB1, whereas CSPG4 has been proposed to mediate the binding/entry in the intestinal sub-epithelial myofibroblasts [30,33,34,56]. How TcdB2 damages the intestinal epithelial layer remains unclear, yet *ex vivo* studies showed that TcdB2 was less toxic than TcdB1 to the colonic organoids [34].

Using the mouse intrarectal instillation model, we found that TcdB1-4, when administrated with the same dose in the mouse intrarectal instillation model, cause distinguishable pathological phenotypes on epithelial barrier disruption, oedema, and inflammatory cell infiltration in the mouse colon. TcdB1 is the most potent one and causes the severest overall damage to colonic tissues, which is consistent with the fact that *C*. *difficile* strains harboring TcdB1 are most frequently isolated in the clinic. TcdB2 and TcdB3 cause similar levels of overall damage to colonic tissues, while TcdB4 only caused mild damages. TcdB1 and TcdB2 differ mainly on FZD-binding. These two toxins induced similar levels of oedema and immune cell infiltration, suggesting that CSPG4-mediated toxin binding and entry into sub-epithelial myofibroblasts is largely responsible for causing these damages. On the other hand, TcdB1 showed severer disruption to the colonic epithelial barrier than TcdB2, which is consistent with the findings that FZD1/2/7 mediate TcdB1 binding and entry into the colonic epithelium. TcdB3 showed lower levels of oedema and higher levels of immune cell infiltration compared with TcdB1 and TcdB2. By using the chimeric toxins, we further defined that these pathologic differences are due to FZD/CSPG4-mediated tissue damages but not distinct substrates of the GTD. TcdB4 only causes mild colonic pathology but is highly potent on HeLa cells, indicating that mouse colonic tissues might not be a suitable target for this toxin variant. Together, these findings reveal key differences in both receptor-binding and damages to colonic tissues for the four major TcdB variants and further validated our classification of TcdB into these subtypes [15].

The disease progression and symptoms of *C*. *difficile* infections *in vivo* could be influenced by expression levels of TcdB, as well as many other factors including but not limited to two other exotoxins TcdA and CDT, toxin expression regulatory factors, sporulation and germination, colonization and adherence factors, fimbriae and pili, and flagella [57–59]. In this study, we mainly focused on investigating the difference among TcdB variants in host receptor recognition and pathology induction. We suggest that the difference of receptor preference among TcdB variants would have implications for the pathogenesis of CDAD. Understanding the functional and pathological differences among major TcdB subtypes and their molecular basis will be critical to the development of effective therapeutic approaches and clinical care in consideration of growing TcdB variations in clinical isolates.

## Materials and methods

### Ethics statement

The intrarectal instillation of TcdB in mice was performed in accordance with institutional guidelines. All animal procedures reported herein were approved by the Institutional Animal Care and Use Committee at Westlake University (IACUC Protocol #19-010-TL). To minimize the distress and pain, the mice were monitored every hour. Animals with signs of pain or distress such as labored breathing, inability to move after gentle stimulation, or disorientation were euthanized immediately. This method was approved by the IACUC and monitored with a qualified veterinarian.

### Materials

HeLa (H1, CRL-1958) and HEK293T (CRL-3216) cells were originally obtained from ATCC. They tested negative for mycoplasma contamination and were authenticated via STR profiling (Shanghai Biowing Biotechnology Co. LTD, Shanghai, China). HeLa-Cas9, HeLa-Cas9 *CSPG4*^*–/–*^, and HeLa-Cas9 *FZD1/2/7*^*–/–*^, HeLa-Cas9 FZD7^CRD^-GPI cells were laboratory stocks and generated as previously described [30,33]. 1D4-tagged Fzd1-10 constructs (in pRK5 vector) were originally generated in Nathans’ laboratory and were obtained from Addgene. The following antibodies were purchased from the indicated vendors: rabbit monoclonal IgG against β-actin (EPR16769) from Abcam, mouse monoclonal IgG against 1D4-tag (MA1-722) from Thermo-Fisher Scientific, and chicken polyclonal IgY against TcdB (#754A) from List Biological Labs. Statistical analysis was performed using OriginPro 8 (v8.0724, OriginLab) and SPSS (ver. 22, IBM) software.

### The *tcdB* genes and cloning

Genes encoding TcdB1 (reference sequence: TcdB_630_), TcdB2 (reference sequence: TcdB_CD196_), TcdB3 (reference sequence: TcdB_1470_), and TcdB4 (reference sequence: TcdB_8864_) were codon-optimized, synthesized by Genscript (Nanjing, China), and cloned into a modified pHT01 vector. The DNA fragments encoding TcdB1^1-2098^, TcdB1^544-2098^, and TcdB1^1285-2366^ were PCR amplified and cloned into the pET28a vector with a His6 tag introduced to their N-terminus.

### Creating chimeric and mutant TcdB constructs

The chimeric toxins were generated with a Gibson Assembly Master Mix (E2611, New England Biolabs) following the manufacturer’s instructions. Mutations in TcdB were generated by site-directed quick-change mutagenesis (Agilent Technologies). All constructs were validated by DNA sequencing.

### Expression, and purification of recombinant TcdB proteins

Recombinant full-length TcdB proteins were expressed in *Bacillus subtilis* SL401 and purified as previously described [15]. In brief, expression was induced with 1 mM isopropyl-β-D-thiogalactoside (IPTG) at 25°C for 16 hours. TcdB1^1-2098^, TcdB1^544-2098^, and TcdB1^1285-2366^ were expressed in *E*. *coli* BL21 (DE3) and purified as His6-tagged proteins. *E*. *coli* cells were cultured at 37°C till OD_600_ reached 0.8. Expression was induced with 0.5 mM IPTG at 16°C overnight. Proteins were purified by Ni-affinity chromatography and followed by Superdex-200 (GE Healthcare) size-exclusion chromatography.

### Generating HeLa-Cas9 FZD7^CRD^-GPI/*CSPG4*^*–/–*^cells

To generate HeLa-Cas9 FZD7^CRD^-GPI/*CSPG4*^*–/–*^cells, the sgRNA sequence targeting *CSPG4* (5’-CCGGAGACACGGAGCAGTGG-3’) was cloned into LentiGuide-puro vector (Addgene). HeLa-Cas9 *FZD7*^*CRD*^ cells were transfected with lentivirus that expresses the sgRNA targeting the *CSPG4* gene. The deficiency of CSPG4 in the selected cells was validated by immunoblot with an anti-CSPG4 antibody (EPR9195, Abcam).

### Cytopathic cell-rounding assay

The cytopathic effect of TcdB variants was analyzed using the gold-standard cell-rounding assay. In brief, cells were exposed to TcdB1, TcdB2, TcdB3, TcdB4, or their derivates for 12–14 hours. The phase-contrast images of cells were recorded (Olympus IX73, ×10–20 objectives). The numbers of round-shaped and normal-shaped cells were counted manually. The percentage of round-shaped cells was analyzed using the Origin software.

### TcdB cell-surface binding assay

The cells were seeded in 24-well plates (Corning Costar) and cultured to ~90% confluence. Binding of toxins to cell-surface was performed by exposing cells to TcdB or TcdB mutants for 10 minutes at room temperature. Cells were washed three times with PBS and then either harvested with RIPA lysis buffer (#P0013D, Beyotime) for immunoblot analysis or fixed with 4% paraformaldehyde for immunofluorescent analysis. For immunofluorescent analysis, TcdB and TcdB mutants were labeled with NHS-Rhodamine (#46406, Thermo Fisher Scientific) following the manufacturer’s instructions.

### 3D structure modeling of TcdB proteins

The modeled 3D structures of TcdB1 and TcdB1^G624T/G626S^ were built based on the full-length TcdB3 crystal structure (PDB 6OQ5) using HHPRED with MODELLER interface (https://toolkit.tuebingen.mpg.de/tools/hhpred) and then aligned using Pymol software.

### Mouse intrarectal instillation assay

BALB/c mice (6–8 weeks, female, specific-pathogen-free) were purchased from the Laboratory Animal Resources Center at Westlake University (Hangzhou, China). Mice were housed in specific-pathogen-free conditions with free access to drinking water and food during the experiments. Mice were anesthetized with pentobarbital sodium. One-millimeter diameter tube was inserted into the rectum (~ 4 cm) and injected 100 μL of saline with or without 10 μg of TcdB. Medical anastomotic glue was used for sealing anal to prevent toxin leakage. After eight hours, mice were euthanized, and their colons were dissected out for histological analysis.

### Histochemistry analysis

Colons from mice challenged with the intrarectal instillation assay were dissected out, fixed by formalin, and embedded with paraffin. Sample blocks were cut into 4 μm thick sections and subjected to H&E and AB/PAS staining. The stained sections were scored blinded by two pathologists based on oedema, inflammatory cell infiltration, and epithelial barrier disruption on a scale of 0 to 3 (mild to severe). The average scores were plotted on the charts. No statistical methods were used to predetermine the sample size.

## Supporting information

S1 Fig The polyclonal antibody recognizes TcdB1, TcdB2, and TcdB3 with similar levels of sensitivity. The sensitivity of the polyclonal antibody against TcdB towards TcdB1, TcdB2, and TcdB3 was tested by immunoblot analysis.
(TIF)

S2 Fig TcdB1 and TcdB3 have the same potential to bind FZD proteins. (A) Primary sequence alignment of the FZD-binding region (1421–1620) between TcdB1 and TcdB3. The only different residue (1575) is highlighted by red. (B) Illustrated representation of TcdB-FZD2 complex, with TcdB in green and FZD2 in cyan. The position of S1575 in the structure is marked by an arrow.
(TIF)

S3 Fig TcdB1^1285-2366^ loses strong binding to CSPG4 on the cell surface. The surface binding experiment showed that full-length TcdB1 but not TcdB1^1285-2366^ robustly bound to the HeLa WT cells. Both TcdB1 and TcdB1^1285-2366^ are only weakly bound to the CSPG4 knockout cells.
(TIF)

S4 Fig Comparison of the toxicities of TcdB3, TcdB1, and two chimeric TcdB in the HeLa cells. (A) The sensitivities of the HeLa cells to TcdB3 and TcdB3-1.1. (B) The sensitivities of the HeLa cells to TcdB1 and TcdB3-1.2. The percentage of rounded cells were plotted over toxin concentrations. (Error bars indicate mean±s.d., n = 6)
(TIF)

S5 Fig Mutagenesis screens identify residues critical for CSPG4-binding. The sensitivities of the HeLa *CSPG4*^*–/–*^(A) and *FZD1/2/7*^*–/–*^(B) cells to TcdB1 and twenty-one TcdB1 mutants. The fitting curves for mutants ADNGR441-445EENIS, G624T/G626S, and S638D/I639R are marked by arrows. The percentage of rounded cells were plotted over toxin concentrations. (Error bars indicate mean±s.d., n = 6)
(TIF)

S6 Fig ADNGR441-445EENIS may impair the TcdB folding/function. Changes of the relative resistance of mutant ADNGR441-445EENIS compared to the WT TcdB1 in the HeLa *CSPG4*^*–/–*^and *FZD1/2/7*^*–/–*^cells were shown in a bar chart. (Error bars indicate mean±s.d., n = 6, n.s. = not significant)
(TIF)

S7 Fig G624T/G626S reduces the binding of TcdB1 to HeLa *FZD1/2/7*^*–/–*^cells. (A) Confocal images showed that fluorescent-labeled TcdB (red) robustly bound to the surface of the HeLa WT cells, while the binding of TcdB1^G624T/G626S^ was significantly reduced. The nuclei were stained by DAPI (blue). DIC, differential interference contrast. Scale bar, 50 μm. (B) Changes of the relative resistance of mutants G624T and G626S compared to the WT TcdB1 in the HeLa *CSPG4*^*–/–*^and *FZD1/2/7*^*–/–*^cells were shown in a bar chart. (Error bars indicate mean±s.d., n = 6, *P<0.001 versus TcdB1, student’s *t-*test).
(TIF)

S8 Fig Overlay of modeled 3D structures of TcdB1 and TcdB1^G624T/G626S^. Overlay of modeled 3D structures of TcdB1 (green) and TcdB1^G624T/G626S^ (yellow) with zoomed-in views containing T/G624, S/G626, Y1824, and N1839. The side chains of T624, S626, Y1824, and N1839 are highlighted.
(TIF)

S1 File The numerical data used in all figures. Excel spreadsheet containing, in separate sheets, the underlying numerical data and statistical analysis for figure panels 1A, 1B, 1C, 1D, 1E, 2B, 3B, 3G, 3H, 3I, 4B, 4D, 5A, 5B, 6B, 6C, 6D, 6E, 7B, 7C, 7D, S4A, S4B, S5, S6, and S7B.
(XLSX)

S2 File A certificate of cell line authentication. (PDF)
