# Supplementary figures and images for: Functional analyses of epidemic Clostridioides difficile toxin B variants reveal their divergence in utilizing receptors and inducing pathology
Source: PLoS Pathog. 2021 Jan 28;17(1):e1009197. doi: 10.1371/journal.ppat.1009197 (PMC7842947; doi:10.1371/journal.ppat.1009197)

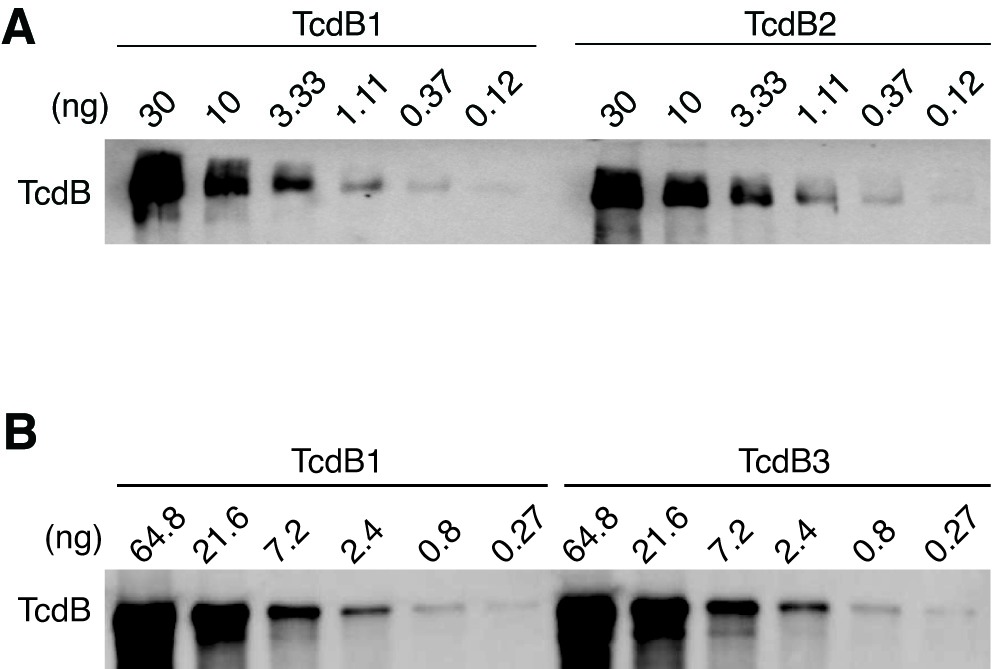

Supplement: S1 Fig — The sensitivity of the polyclonal antibody against TcdB towards TcdB1, TcdB2, and TcdB3 was tested by immunoblot analysis. (TIF) [file ppat.1009197.s001.tif]

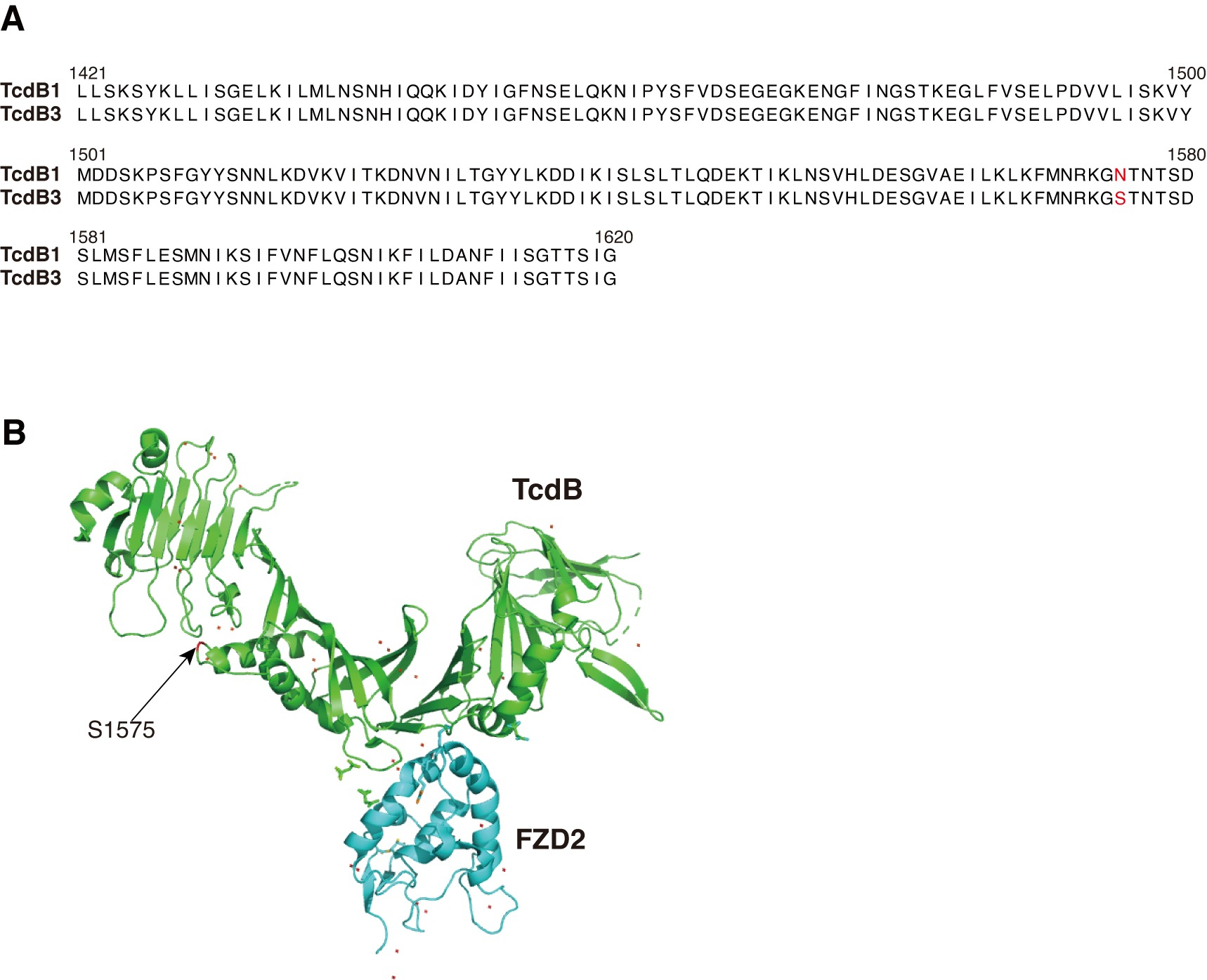

Supplement: S2 Fig — (A) Primary sequence alignment of the FZD-binding region (1421–1620) between TcdB1 and TcdB3. The only different residue (1575) is highlighted by red. (B) Illustrated representation of TcdB-FZD2 complex, with TcdB in green and FZD2 in cyan. The position of S1575 in the structure is marked by an arrow. (TIF) [file ppat.1009197.s002.tif]

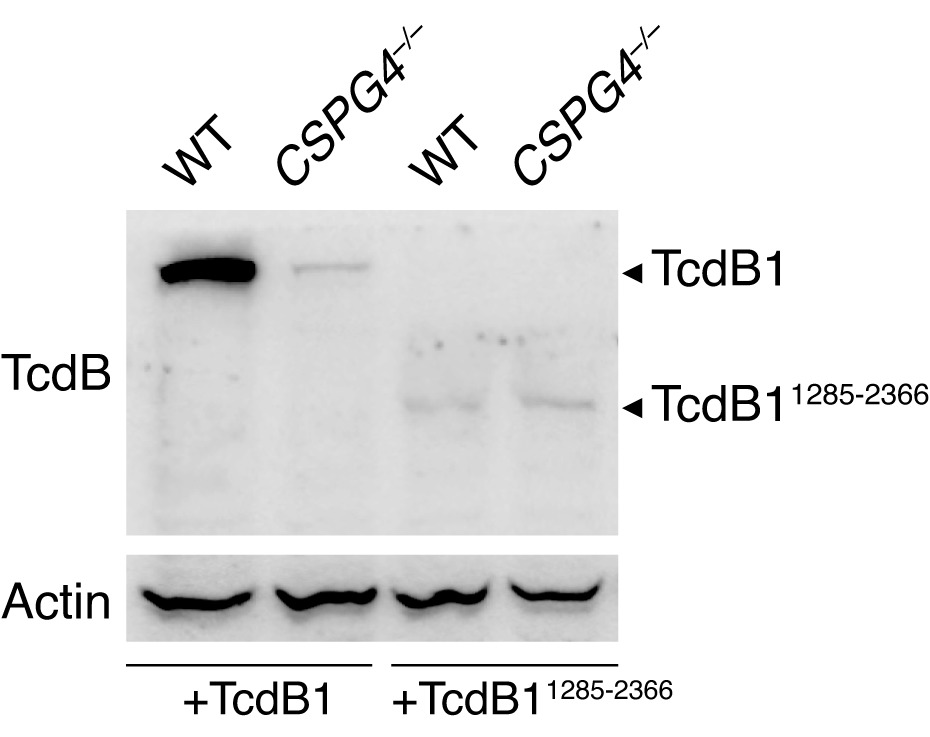

Supplement: S3 Fig — The surface binding experiment showed that full-length TcdB1 but not TcdB11285-2366 robustly bound to the HeLa WT cells. Both TcdB1 and TcdB11285-2366 are only weakly bound to the CSPG4 knockout cells. (TIF) [file ppat.1009197.s003.tif]

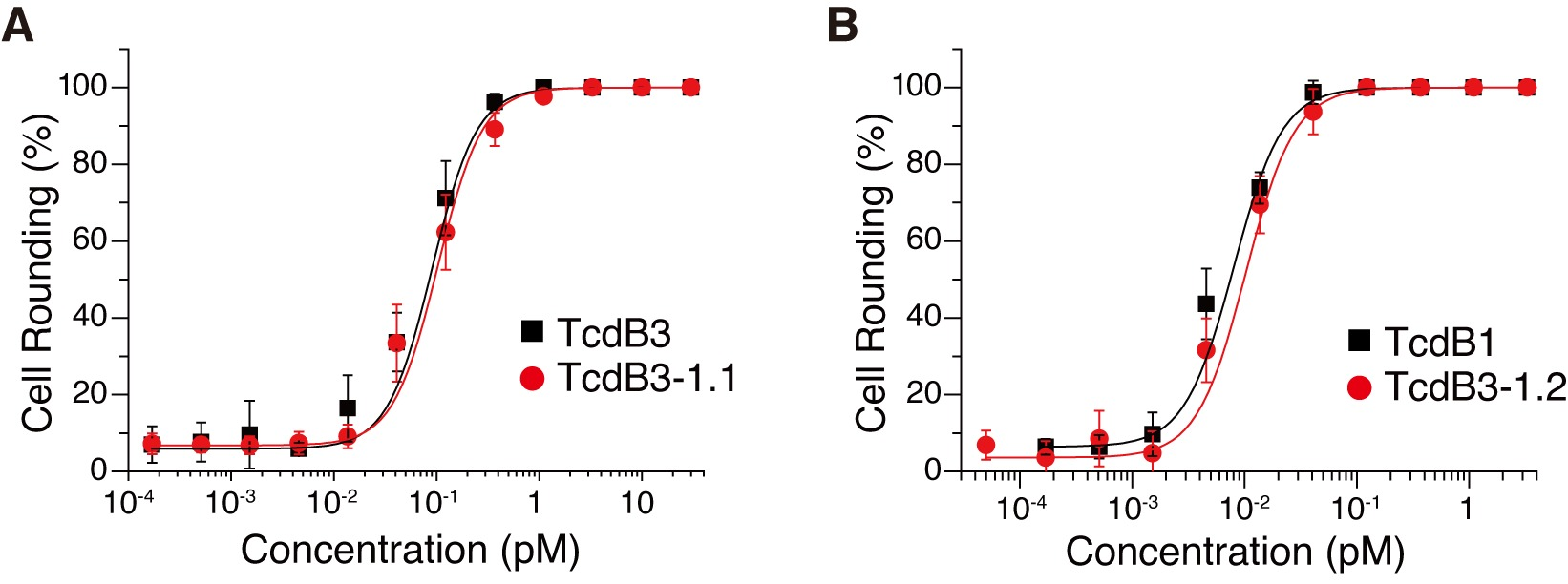

Supplement: S4 Fig — (A) The sensitivities of the HeLa cells to TcdB3 and TcdB3-1.1. (B) The sensitivities of the HeLa cells to TcdB1 and TcdB3-1.2. The percentage of rounded cells were plotted over toxin concentrations. (Error bars indicate mean±s.d., n = 6) (TIF) [file ppat.1009197.s004.tif]

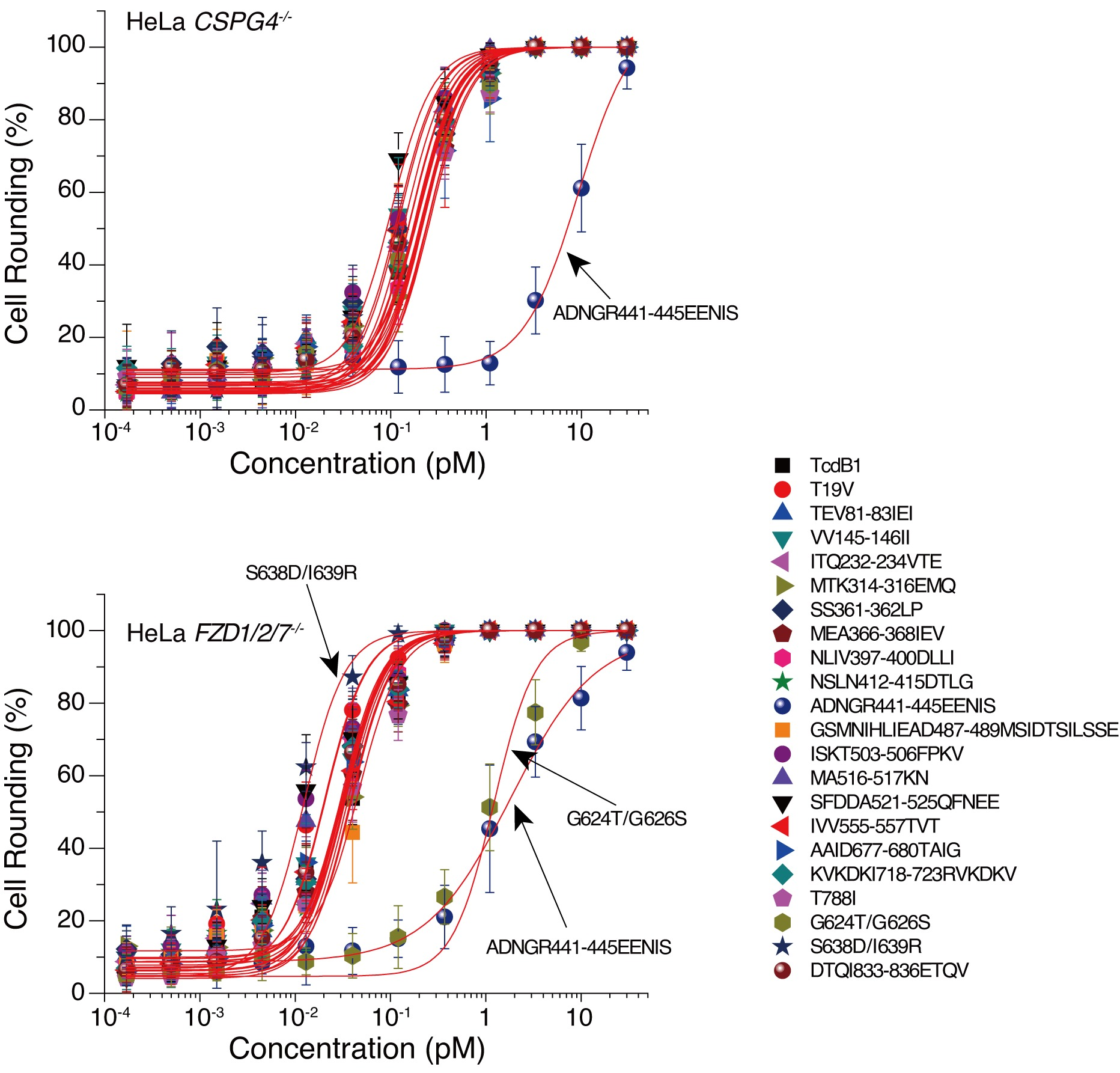

Supplement: S5 Fig — The sensitivities of the HeLa CSPG4–/–(A) and FZD1/2/7–/–(B) cells to TcdB1 and twenty-one TcdB1 mutants. The fitting curves for mutants ADNGR441-445EENIS, G624T/G626S, and S638D/I639R are marked by arrows. The percentage of rounded cells were plotted over toxin concentrations. (Error bars indicate mean±s.d., n = 6) (TIF) [file ppat.1009197.s005.tif]

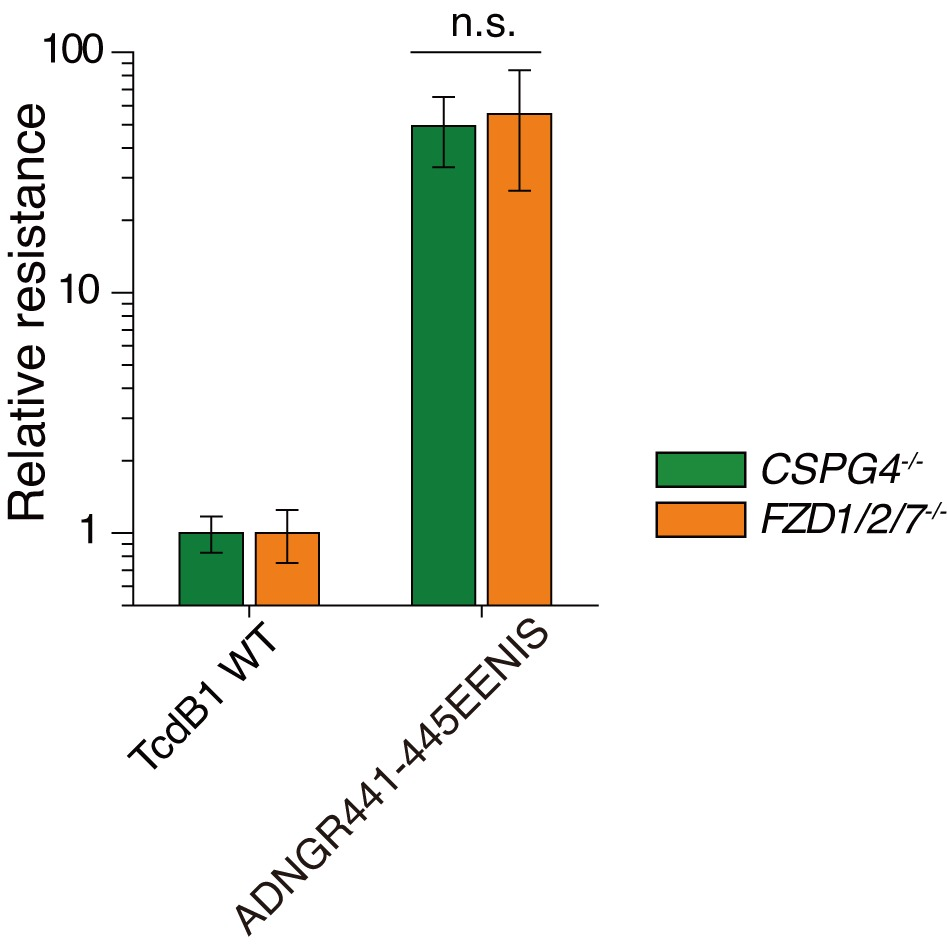

Supplement: S6 Fig — Changes of the relative resistance of mutant ADNGR441-445EENIS compared to the WT TcdB1 in the HeLa CSPG4–/–and FZD1/2/7–/–cells were shown in a bar chart. (Error bars indicate mean±s.d., n = 6, n.s. = not significant) (TIF) [file ppat.1009197.s006.tif]

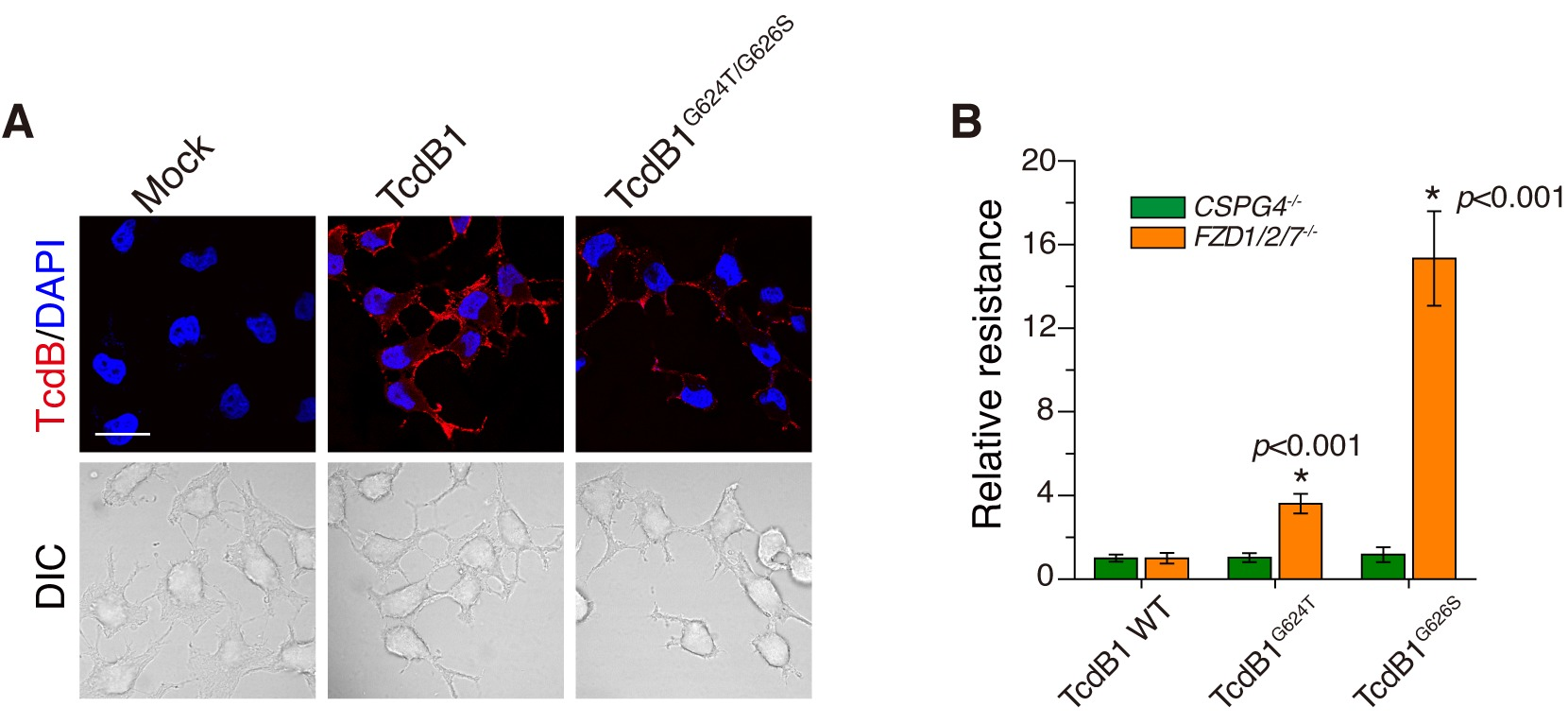

Supplement: S7 Fig — (A) Confocal images showed that fluorescent-labeled TcdB (red) robustly bound to the surface of the HeLa WT cells, while the binding of TcdB1G624T/G626S was significantly reduced. The nuclei were stained by DAPI (blue). DIC, differential interference contrast. Scale bar, 50 μm. (B) Changes of the relative resistance of mutants G624T and G626S compared to the WT TcdB1 in the HeLa CSPG4–/–and FZD1/2/7–/–cells were shown in a bar chart. (Error bars indicate mean±s.d., n = 6, *P<0.001 versus TcdB1, student’s t-test). (TIF) [file ppat.1009197.s007.tif]

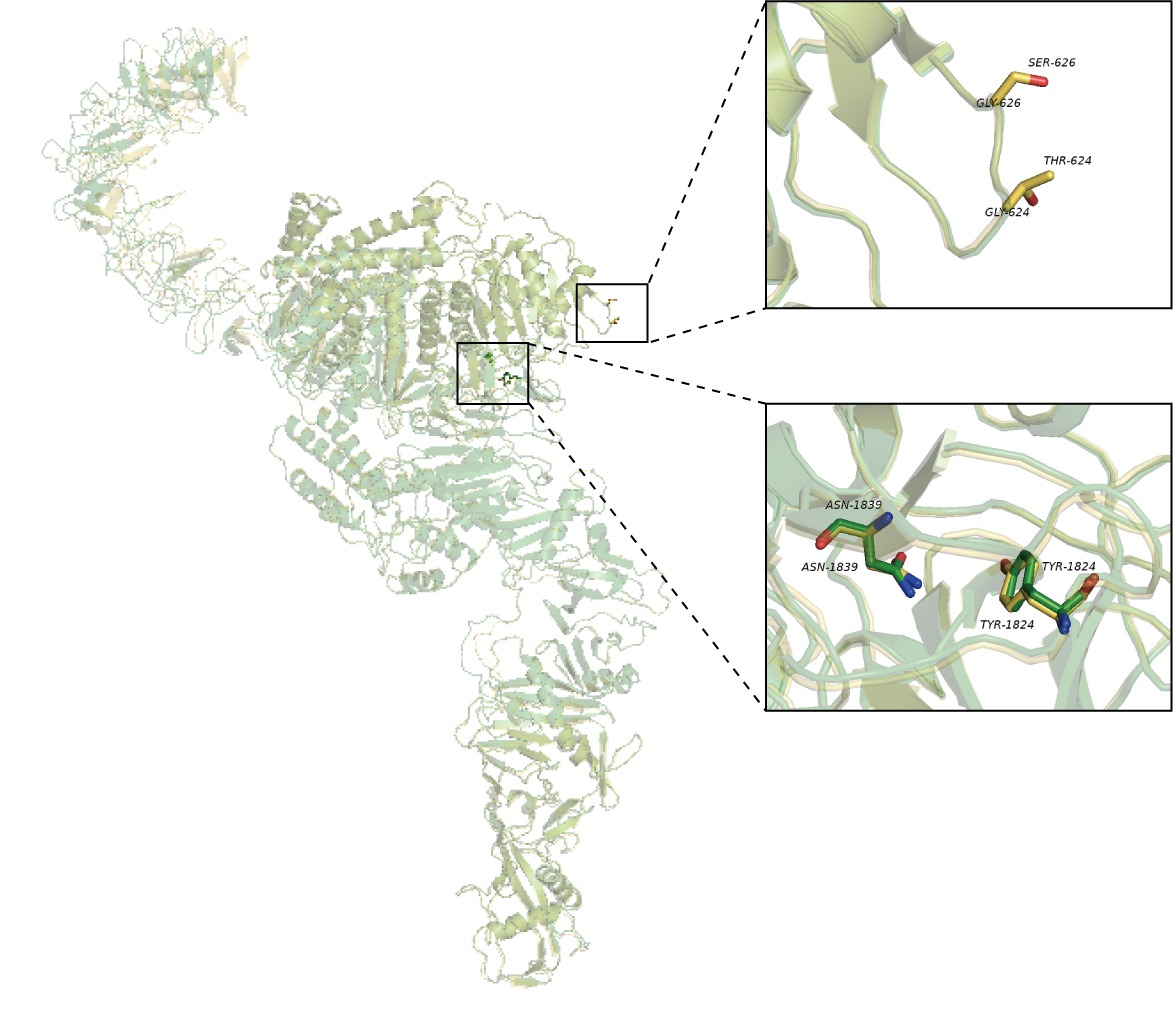

Supplement: S8 Fig — Overlay of modeled 3D structures of TcdB1 (green) and TcdB1G624T/G626S (yellow) with zoomed-in views containing T/G624, S/G626, Y1824, and N1839. The side chains of T624, S626, Y1824, and N1839 are highlighted. (TIF) [file ppat.1009197.s008.tif]
